# Expression of cytokine-mRNA in PBMC of HIV-1 subtype C infected individuals with opportunistic viral infections from South India

**DOI:** 10.1186/1471-2334-14-S3-E10

**Published:** 2014-05-27

**Authors:** Jaiprasath Sachithanandham, Veena Vadhini Ramalingam, Jaganathan Raja, Ooriyapadickal Cherian Abraham, Susanne A Pulimood, Rajesh Kannangai

**Affiliations:** 1 Departments of Clinical Virology, Christian Medical College, Vellore, India; 2 Internal Medicine, Christian Medical College, Vellore, India; 3 Dermatology, Christian Medical College, Vellore, India

## Background

HIV disease progression is associated with marked change in the level of plasma cytokines. The study reported here investigated the level of mRNA expression of different cytokines: TNF alpha, INF-gamma, IL-10 and IL-21 in the peripheral blood mononuclear cells (PBMC) among the HIV infected individuals with viral opportunistic infections.

## Methods

We quantified the mRNA levels of 4 cytokines in PBMC directly from the blood of individuals infected with HIV and normal healthy controls using TaqMan based RealTime PCR.

## Results

The mRNA expressions of all the 4 cytokines in HIV-1 infected individuals were significantly higher compared to healthy controls (*p* value range 0.0004-0.01). The mean level of IL-10, INF-gamma and TNF alpha were higher in HIV infected individuals with low CD4 counts (<300 cells/µL). The IL-10 expression showed a significant negative correlation with CD4 counts (*r* = -0.25, *p* = 0.04) while IL-21 showed a positive correlation with CD4 counts (*r* = 0.26, *p* = 0.03). There was significant negative correlation between the CMV viral load and IL-21 expression.

## Conclusion

The study showed a significant negative correlation between CD4+ T cell counts and IL-10 mRNA expression showing a shift towards TH2 profile during the late stages of the HIV disease in subtype C infected individuals. This study on HIV-1 subtype C infected individuals with opportunistic viral infections like EBV and CMV adding new information in such specific opportunistic infections.

